# A Comparative Analysis of Gene and Protein Expression Throughout a Full 28-Day Retinal Regeneration Time-Course in Adult Zebrafish

**DOI:** 10.3389/fcell.2021.741514

**Published:** 2021-11-01

**Authors:** Ashley C. Kramer, Katherine Gurdziel, Ryan Thummel

**Affiliations:** ^1^Department of Ophthalmology, Visual and Anatomical Sciences, Wayne State University School of Medicine, Detroit, MI, United States; ^2^Genome Sciences Core, Wayne State University, Detroit, MI, United States

**Keywords:** Müller glia, stem cell, gliosis, 3′RNA-seq, regeneration, retina

## Abstract

Following photoreceptors ablation by intense light exposure, adult zebrafish are capable of complete regeneration due to the ability of their Müller glia (MG) to re-enter the cell cycle, creating progenitors that differentiate into new photoreceptors. The majority of previous reports on retinal regeneration focused on the first few days of the regenerative response, which include MG cell-cycle re-entry and progenitor cell proliferation. With this study, we analyzed the full 28-day time-course of regeneration by pairing a detailed morphological/immunological analysis with RNA-seq transcriptional profiling at 8 key time points during retinal regeneration. We observed several novel findings. First, we provide evidence for two separate peaks of MG gliosis, with the secondary gliotic peak occurring after MG cell-cycle re-entry. Second, we highlight a distinct transcriptional shift between 5- and 10-days post lesion that highlights the transition from progenitor proliferation to differentiation into new photoreceptors. Third, we show distinctly different patterns of transcriptional recovery of the photoreceptor opsins at 28 days post lesion. Finally, using differential gene expression analysis, we revealed that the established functional recovery of the retina at 28 days post lesion does not, in fact, return to an undamaged transcriptional state, potentially redefining what the field considers complete regeneration. Together, to our knowledge, this work represents the first histological and transcriptomic map of a 28-day time-course of retinal regeneration in adult zebrafish.

## Introduction

According to the CDC, there are currently over 12 million Americans suffering from vision loss due to diseases affecting the retina, including diabetic retinopathy, glaucoma, and age-related macular degeneration (Wittenborn et al., 2013). To date, therapeutic attempts to replace lost retinal neurons have been met with limited success, and the process of true retinal regeneration remains elusive to mammals (Hamon et al., 2016; Yao et al., 2018). Following retinal damage in mammals, Müller glia (MG) of the retina undergo an acute gliosis, activating various cytoskeletal and neuroprotective gene pathways and releasing neurotrophic factors and free radical scavengers (Bringmann et al., 2009; Hippert et al., 2015). This initial damage response is neuroprotective to retinal neurons. However, if this acute event persists into a chronic gliosis, significant retinal damage can occur due to loss of normal MG function, retinal remodeling, and glial scar formation (Bringmann et al., 2009). In contrast to mammals, the zebrafish exhibits profound retinal regenerative capacity. For example, in response to phototoxic degeneration of rod and cone photoreceptors, zebrafish MG undergo an asymmetric cell division, generating one Müller glia-derived progenitor cell (MGPC) and one MG that retains its innate glial function (Nagashima et al., 2013). The MGPC daughter cell divides symmetrically to generate pools of multipotent progenitor cells. These cells subsequently migrate along the MG toward the outer retina and differentiate into new rod and cone photoreceptors. Functional recovery of vision occurs by 28 days post lesion, a time point largely used by the field as complete regeneration (Ramachandran et al., 2011; Wan et al., 2012; Nelson et al., 2013; Taylor et al., 2015; Wan and Goldman, 2017).

The majority of studies on zebrafish retinal regeneration have focused on individual signaling pathways during the first few days post lesion, when MG re-enter the cell cycle and the pools of multipotent progenitor cells form. Using pharmacological and/or genetic approaches, multiple important findings have elucidated key pathways that are critical for the regenerative process, including *wnt/beta-catenin, notch, tnf*, and *fgf* (Ramachandran et al., 2011; Nelson et al., 2013; Wan and Goldman, 2017). The first unbiased approach to understanding the gene networks that regulate retinal regeneration was described in 2007, in which a microarray was performed at 6 early time points post phototoxic lesion of the retina (Kassen et al., 2007). More recently, a 2020 study utilized single-cell RNA sequencing to perform a cross-species comparison of the MG response to retinal damage in zebrafish, chick and mouse (Hoang et al., 2020). Both of these studies focused only on early time points in retinal degeneration/regeneration. To date, no study has paired morphological/immunological changes with transcriptional profiling throughout the full 28-day time-course.

In this study, we paired 3′mRNA-seq with immunohistochemistry to investigate the process of zebrafish retinal regeneration following phototoxic lesion throughout the entire 28-day time-course. Whole retinal tissue was collected from eyes at the following eight key time points during the regenerative process: 0 h (no damage), 24 hpl (peak damage/MG gliosis), 36 hpl (initiation of MG cell cycle re-entry), 72 hpl (peak proliferation of MGPCs), 5 dpl (initiation of photoreceptor differentiation), 10 dpl (presumptive completion of photoreceptor differentiation), 14 dpl (photoreceptor outer segment growth), and 28 dpl (complete functional regeneration) (Vihtelic and Hyde, 2000; Kassen et al., 2007; Thummel et al., 2008a; Thomas et al., 2012; Lenkowski and Raymond, 2014). At each of these time points, retinas from the left eyes of each animal were harvested for 3′mRNA-seq as individual biological replicates, and whole right eyes were collected for immunohistochemistry. We report several novel findings throughout the time-course. We provide evidence for a secondary gliotic peak of the MG following cell-cycle re-entry, and highlight a distinct window of time between 5- and 10-days post lesion as a transition from progenitor proliferation to differentiation into new photoreceptors. Lastly, we discovered distinctly different patterns of transcriptional recovery at 28 dpl, despite morphological and functional recovery. Together, these new data provide the field with an updated morphological and transcriptomic map of the complete 28-day cycle of retinal degeneration and regeneration in adult zebrafish and raise new avenues for exploration of key stages of this process.

## Results

### Expression Patterns Revealed by 3′mRNA-Seq Closely Mimic the Expression of Previously Established Genetic Markers During Retinal Regeneration

**FIGURE 1:**
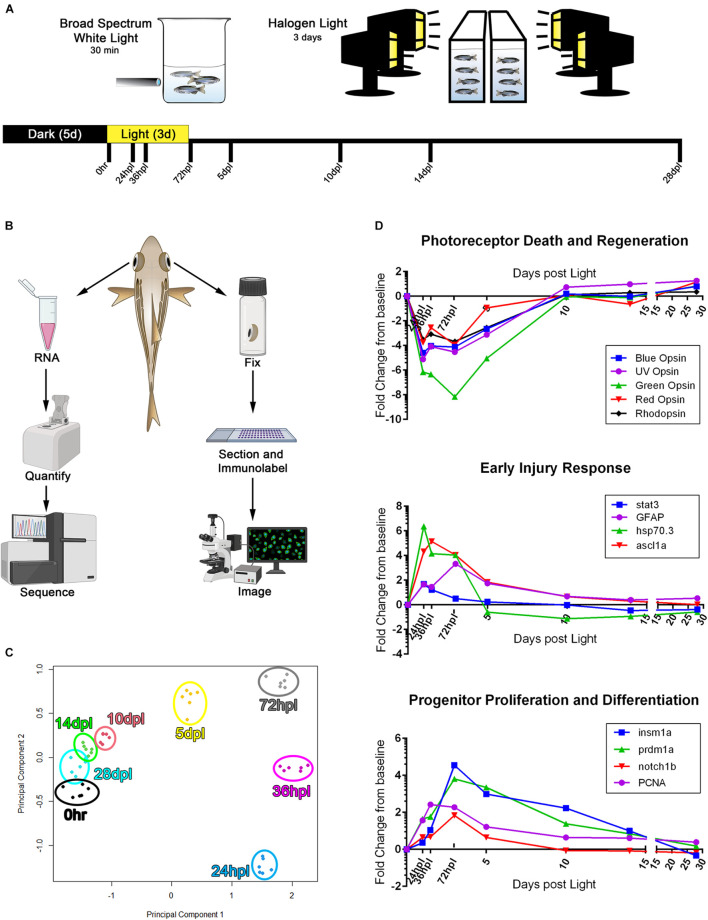
3′mRNA-seq analysis following phototoxic lesion and regeneration confirms gene expression trends known in the field. **(A)** Experimental design used for phototoxic lesion, with tissue collection time-points along the bottom designated in hours post-light (hpl) and days post-light (dpl). **(B)** Schematic demonstrating the workflow for tissue collection for each individual fish. **(C)** Principle component analysis constructed using the top 200 genes differentially regulated as compared to the 0 h baseline. All genes used in the analysis had an FDR < 7.5 × 10^− 5^. **(D)** RNA expression fold change from 0 h baseline for three processes of retinal regeneration well described in the literature, demonstrating validation of the 3′mRNA-seq method in determining gene expression changes during the retinal regeneration process. The genes highlighted represent three commonly reported stages in the regeneration process including the degeneration and regeneration of photoreceptors, early responding genes that turn on within 24 hpl, and progenitor cell response genes which peak around 72 hpl. Regeneration of lost photoreceptors is evidenced by the re-emergence of opsin gene expression starting at 5 dpl. For data presented in **(C,D)**, *n* = 6 biological replicates. With the exception of the zebrafish rendering, all clipart in **(B)** was downloaded from BioRender.com.

We utilized 3′mRNA sequencing (Ma et al., 2019) paired with a detailed morphological and immunological analysis at eight key time points over a 28-day period (Figure 1A). We chose previously characterized time points in an effort to cross reference this methodology for accuracy and precision (Vihtelic and Hyde, 2000; Kassen et al., 2007; Thummel et al., 2008a; Thomas et al., 2012; Lenkowski and Raymond, 2014). For each animal, the right eye was used for immunohistochemistry (IHC) and the isolated retina of the left eye was designated for transcriptional profiling using 3′mRNA-seq (Figure 1B). To determine whether distinct differences were apparent in the transcriptomes of the retinas for each time point, we performed a principal component analysis (PCA) using the top 200 differentially regulated genes, all which had an FDR < 0.05. We found that each condition tightly clustered among the six biological replicates for each time point (Figure 1C). The early time points in the regeneration time-course (24, 36, and 72 hpl) showed the most distinct transcription profiles, clustered farthest away from the undamaged 0 h reference group, and as the regeneration time-course progressed, the gene expression profiles clustered closer to the 0 h group (Figure 1C).

Next, we plotted normalized gene expression over the entire time-course for select gene sets commonly reported in the field: photoreceptor destruction and regeneration, early injury response, and stem-cell processes (Figure 1D). Within the first 24 h of phototoxic lesion, the photoreceptors undergo significant damage, resulting in all opsin transcript levels decreasing to well below the 0 h baseline (*opn1sw1, opn1sw2, opn1mw1-4, opn1lw1-2*, and *rho*; Figure 1D). Phototoxic damage generated a peak of apoptotic photoreceptor cells in the outer retina from 24 to 36 hpl, as confirmed by TUNEL assay (Supplementary Figure 1), but left the inner retinal neurons intact throughout the time-course (Supplementary Figure 2). The destruction of photoreceptors resulted in early elevation (24–72 hpl) of several genes involved in injury signaling and gliosis (*stat3, GFAP, hsp70.3, ascl1a*) (Figure 1D; Zhang et al., 2005; Bernardos and Raymond, 2006; Bernardos et al., 2007; Kassen et al., 2007; Qin et al., 2009; Craig et al., 2010; Ramachandran et al., 2010; Nelson et al., 2012, 2013). Following this early stress response, we observed an upregulation of progenitor proliferation and differentiation genes critical for regeneration pathways (Figure 1D), including *insm1a, prdm1a, notch1b, PCNA* (Vihtelic and Hyde, 2000; Raymond et al., 2006; Thummel et al., 2008b, 2011; Brzezinski et al., 2010; Forbes-Osborne et al., 2013; Campbell et al., 2020). Lastly, *rhodopsin*, and the UV, Blue, Green, and Red cone opsins began to recover as soon as 5 dpl, continued to increase, and eventually surpassed the dark-adapted 0 h control levels (Figure 1D). Together, this initial analysis suggested that the transcriptional changes we observed recapitulated previously reported genetic profiles in the literature and gave us confidence in pursuing a deeper analysis of the dataset.

### Comparative Analysis and Quantification of Transcription and Protein Levels Demonstrates Dynamic Changes to Cone Photoreceptor Opsins During the 28-Day Time-Course

**FIGURE 2:**
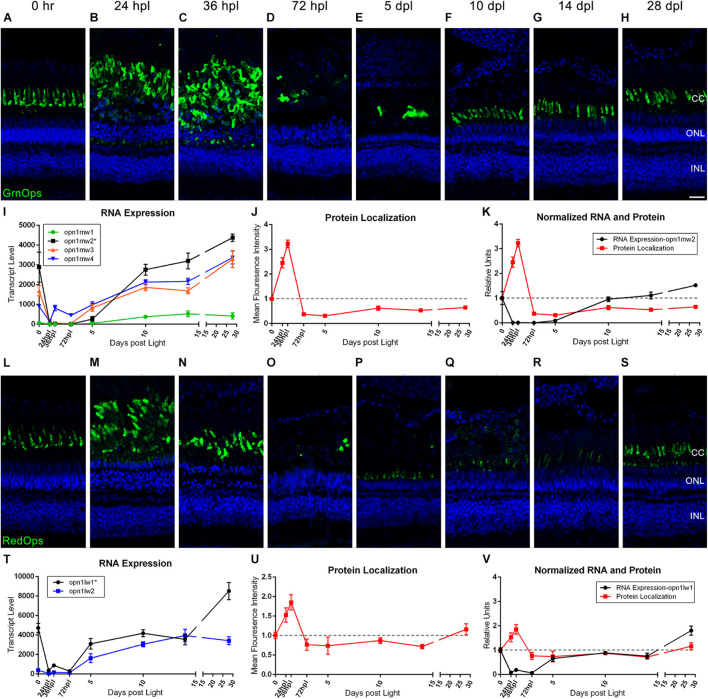
Green and red cone photoreceptor morphology paired with gene expression of isoforms throughout a 28 day lesion and regeneration time-course. **(A–H)** Green cone photoreceptor degeneration and regeneration is demonstrated in these retinal sections collected at baseline (0 h) through 28 days post phototoxic lesion (dpl), hours post light are denoted (hpl). Sections were immunolabeled with anti-Green Opsin and nuclei were stained blue with TO-PRO-3. Cone photoreceptors are mostly destroyed at 72 hpl after a period of initial hypertrophy (*n* = 5–6). **(I)** Graph of transcript pseudo-counts for all 4 paralogs of Green Opsin from 3′mRNA-seq of individual adult zebrafish retinas for each timepoint (*n* = 6). **(J)** ImageJ pixel intensity quantification for the Green Opsin signal in the confocal images normalized to 1 demonstrating relative intensity of protein localization within the retina at each timepoint. **(K)** Overlay of RNA expression for the most highly expressed Green Opsin, *opn1mw2*, normalized to 1 and ImageJ protein localization normalized to one. **(L–S)** Red cone photoreceptor degeneration and regeneration. Sections were immunolabeled with anti-Red Opsin and nuclei were stained blue with TO-PRO-3 (*n* = 5–6). **(T)** Graph of transcript pseudo-counts for both paralogs of Red Opsin from 3′mRNA-seq of individual adult zebrafish retinas for each timepoint (*n* = 6). **(U)** ImageJ pixel intensity quantification for the Red Opsin signal in the confocal images normalized to 1 demonstrating relative intensity of protein localization within the retina. **(V)** Overlay of RNA expression for the most highly expressed Red Opsin, *opn1lw1*, normalized to 1 and ImageJ protein localization normalized to one. Asterisks in paralog keys in **(I,T)** represent the most dominantly expressed paralog at the 0 h baseline that was also graphed in the merged normalized graphs **(K)** and **(V)**. Scale bar represents 5 μm.

**FIGURE 3:**
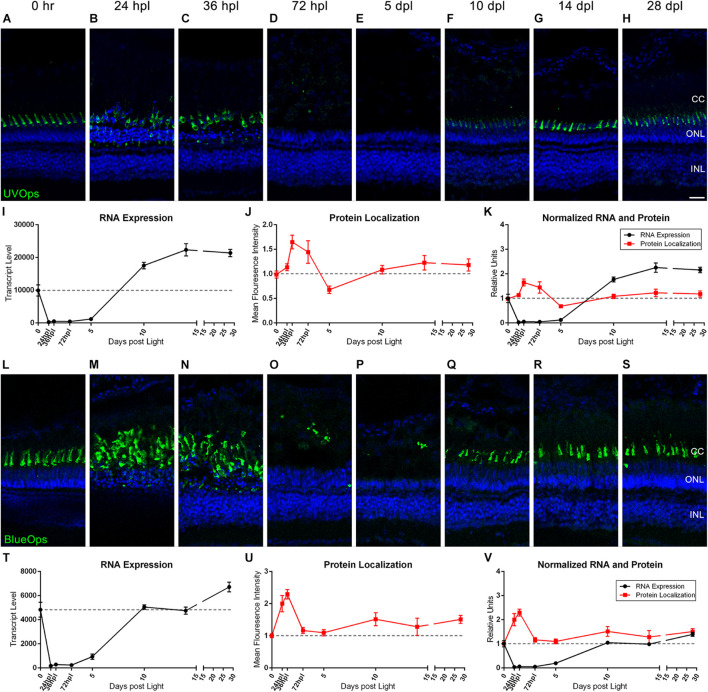
UV and Blue cone photoreceptor morphology paired with gene expression throughout a 28 day lesion and regeneration time-course. **(A–H)** UV cone photoreceptor degeneration and regeneration is demonstrated in these retinal sections collected at baseline (0 h) through 28 days post phototoxic lesion (dpl), hours post light are denoted (hpl). Sections were immunolabeled with anti-UV Opsin and nuclei were stained blue with TO-PRO-3. Cone photoreceptors are mostly destroyed at 72 hpl after a period of initial hypertrophy (*n* = 5–6). **(I)** Graph of transcript pseudo-counts for UV Opsin (*opn1sw1*) from 3′mRNA-seq of individual adult zebrafish retinas for each timepoint (*n* = 6). **(J)** ImageJ pixel intensity quantification for the UV Opsin signal in the confocal images normalized to 1, demonstrating relative intensity of protein localization within the retina. **(K)** Overlay of *opn1sw1* RNA expression normalized to 1 and ImageJ protein localization normalized to one. **(L–S)** Blue cone photoreceptor degeneration and regeneration. Sections were immunolabeled with anti-Blue Opsin and nuclei were stained blue with TO-PRO-3 (*n* = 5–6). **(T)** Graph of transcript pseudo-counts for Blue Opsin (*opn1sw2*) from 3′mRNA-seq of individual adult zebrafish retinas for each timepoint (*n* = 6). **(U)** ImageJ pixel intensity quantification for the Blue Opsin signal in the confocal images normalized to 1, demonstrating relative intensity of protein localization within the retina. **(V)** Overlay of *opn1sw2* RNA expression normalized to 1 and ImageJ protein localization normalized to one. Scale bar represents 5 μm.

Previous and recent reports have characterized the immunolocalization profiles of cone photoreceptor opsins during retinal regeneration (Vihtelic and Hyde, 2000; Hoang et al., 2020), but a direct comparison of mRNA to protein changes throughout the entire regeneration time-course has not been reported. To correlate transcriptomic changes with protein changes, we quantified IHC fluorescence at each time point and overlaid the normalized changes in mean fluorescence intensity (MFI) with the corresponding gene transcripts for all of the cone photoreceptor opsins (Figures 2, 3). Interestingly, protein levels for all four cone opsins increased at 24 and 36 hpl (Figures 2J,U, 3J,U), as the degenerating cones were concentrated in a debris field (Figures 2B,C,M,N, 3B,C,M,N). Starting at 72 hpl, protein levels of all the opsins significantly decreased as the cellular debris was cleared and these levels remained low through 5 dpl (Figures 2D,E,J,O,P, 3D,E,J,O,P). At 10 dpl, expression of all four opsins re-emerged in newly formed cones with short outer segments (Figures 2F,Q, 3F,Q), which generally extended in length and intensity through 28 dpl (Figures 2H,S, 3H,S). When we compared the IHC quantifications with the gene expression changes, cone opsin gene expression dropped significantly in the early time points while the protein quantification had an apparent inverse relationship with gene expression at these early stages (Figures 2K,V, 3K,V). During the later time points, both qualitatively and quantitatively, the protein recovery closely followed gene expression trends.

The Green and Red Opsin genes have multiple paralogs, and our approach gave new insight into the paralog(s) that were most prominently expressed during retinal regeneration. For example, the *opn1mw1* paralog of Green Opsin was not highly expressed (Figure 2I), but all other paralogs (*opn1mw2, opn1mw3*, and *opn1mw4)* were expressed and generally followed the same drop and then recovery of expression throughout the time-course (Figure 2I). Of the two Red Opsin paralogs (*opn1lw1* and *opn1lw2*), the gene expression profile of *opn1lw1* more closely matched the morphological/IHC expression (compare Figures 2L–S,T). Interestingly, however, *opn1lw2* was not highly expressed in undamaged 0 h retinas, but increased over 2000-fold by 10 dpl, and remained high through 28 dpl (Figure 2T). UV Opsin (*opn1sw1)* and Blue Opsin (*opn1sw2*) each have only one paralog and both followed a similar drop and then recovery of expression throughout the time-course; however, the UV Opsin was expressed at a much higher levels compared with all other cone opsins (Figures 3I,T). Finally, the expression of the cone opsins was higher at 28 dpl than at the dark-adapted 0 hpl baseline, which initially suggested an overcompensation of gene expression despite apparent morphological recovery (Figures 2I,T, 3I,T).

### Inflammatory Cell Infiltration Correlates Closely With the Presence of Lesion-Induced Photoreceptor Outer Segment Damage

**FIGURE 4:**
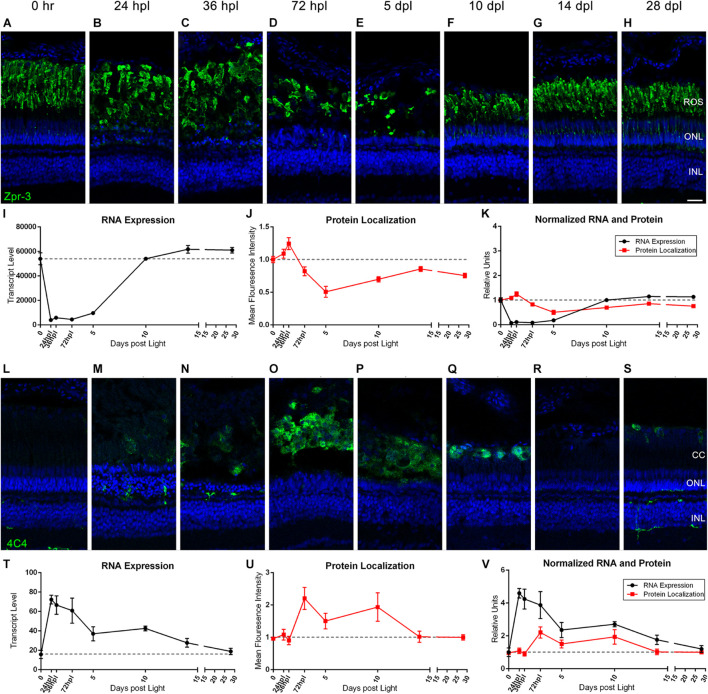
Rod photoreceptors degenerate in coordination with the infiltration of 4C4+ microglia/macrophages. **(A–H)** Rod photoreceptor degeneration and regeneration is demonstrated in these retinal sections collected at baseline (0 h) through 28 days post phototoxic lesion (dpl), hours post light are denoted (hpl). Sections were immunolabeled with Zpr-3, which stains rod photoreceptors and nuclei were stained blue with TO-PRO-3. Rod photoreceptors are notably slower to degenerate than the cone photoreceptors with lowest visual signal by 5 dpl (*n* = 5–6). **(I)** Graph of transcript pseudo-counts for *rhodopsin* (*rho*) from 3′mRNA-seq of individual adult zebrafish retinas for each timepoint (*n* = 6). **(J)** ImageJ pixel intensity quantification for the Zpr-3 signal in the confocal images normalized to 1, demonstrating relative intensity of protein localization within the retina. **(K)** Overlay of *rho* RNA expression normalized to 1 and ImageJ protein localization normalized to one. **(L–S)** Infiltration of microglia/macrophage inflammatory cells into the retina demonstrating a peak infiltration at 72 hpl, corresponding with the drop in protein signal from rod photoreceptors at the same timepoints. Sections were immunolabeled with anti-4C4 which labels microglia/macrophages and nuclei were stained blue with TO-PRO-3 (*n* = 5–6). **(T)** Graph of transcript pseudo-counts for *mpeg1.1*, a gene expressed by macrophages, from 3′mRNA-seq of individual adult zebrafish retinas for each timepoint (*n* = 6). **(U)** ImageJ pixel intensity quantification for the 4C4 signal in the confocal images normalized to 1, demonstrating relative intensity of protein localization within the retina. **(V)** Overlay of *mpeg1.1* RNA expression normalized to 1 and ImageJ protein localization normalized to one. Scale bar represents 5 μm.

During the process of zebrafish retinal regeneration, inflammatory 4C4+ microglia appear in the retina as Zpr-3+ rod photoreceptor outer segments degenerate (Saito et al., 2020; Silva et al., 2020). To directly compare the kinetics of photoreceptor degeneration with immune cell infiltration, we juxtaposed rod photoreceptor IHC and gene expression with microglia IHC and gene expression (Figure 4). First, we found that the destruction and recovery of the rod photoreceptors followed a very similar pattern to the cone photoreceptors. Protein localization of Zpr-3 increased slightly through 36 hpl as degenerating photoreceptor outer segments were concentrated in a debris field (Figures 4B,C,J), but then rapidly decreased through 5 dpl (Figures 4D,E,J). Transcript levels of *rhodopsin* decreased dramatically by 24 hpl (Figure 4I). Of note, *rhodopsin* was the highest expressed opsin in the zebrafish retina, exhibiting gene expression fivefold higher than the most highly expressed cone opsin (Figure 4I). Both protein and transcript levels remained low through 5 dpl, and then steadily recovered through 28 dpl (Figures 4H–K). Next, we observed that the peak increase in 4C4+ microglia in the outer retina at 72 hpl corresponded closely with the clearing of destroyed rod and cone photoreceptor outer segments (compare Figures 3D, 4D with Figures 4O,P). This increase in morphologically activated 4C4+ microglia spanned the time period between 72 hpl and 10 dpl, dropping back down to baseline levels by 14 dpl, when the newly born photoreceptors are maturing. At the transcript level, we analyzed *mpeg1.1* expression, a known transcriptional marker of microglia and macrophages in zebrafish (Ferrero et al., 2020). The rise in *mpeg1.1* expression immediately preceded the peak in 4C4+ microglia localized to the debris field, and then steadily dropped as microglia cleared the area (Figures 4L–V). The presence of 4C4+ microglia at 72 hpl also immediately followed the peak of apoptosis in the outer retina from 24 to 36 hpl as evidenced by TUNEL assay (Supplementary Figure 1). Together, these data support a previous suggestion that quantification of 4C4+ microglia could be used as a proxy for retinal neuron damage (Craig et al., 2008; White et al., 2017; Ranski et al., 2018; Mitchell et al., 2019; Issaka Salia and Mitchell, 2020).

### Characterization of Müller Glia Reactive Gliosis and Proliferation Reveals Novel Observations in GFAP Gene and Protein Kinetics Relative to Stem Cell Function

**FIGURE 5:**
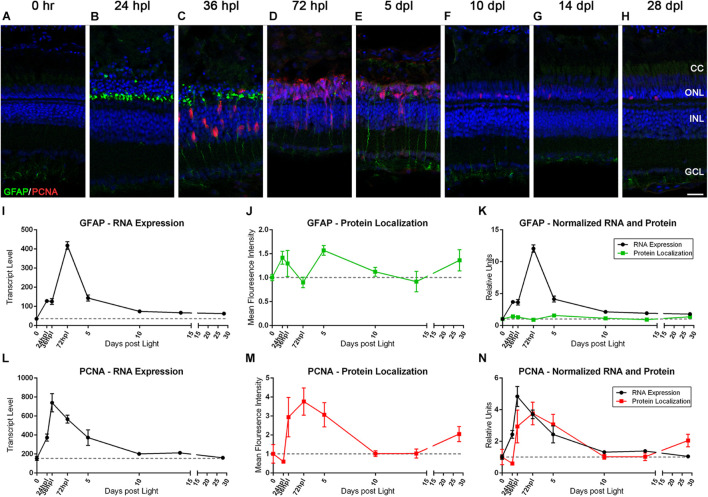
The Müller glia response to phototoxic lesion paired with gene expression signatures. **(A–H)** The Müller glia (MG) response to phototoxic lesion is demonstrated in these retinal sections collected at baseline (0 h) through 28 days post phototoxic lesion (dpl), hours post light are denoted (hpl) by immunolabeling the intermediate filaments of MG with anti-GFAP (green) and their entry into the cell cycle with PCNA (red). Nuclei were stained blue with TO-PRO-3. Gliosis is highlighted in a biphasic manner: in the ONL MG end-feet at 24 hpl, and then spanning the length of MG at 5 dpl. The PCNA localization demonstrates single MG nuclei entering the cell cycle at 36 hpl with peak proliferation of MGPCs at 72 hpl (*n* = 5–6). **(I)** Graph of transcript pseudo-counts for *GFAP* from 3′mRNA-seq of individual adult zebrafish retinas for each timepoint (*n* = 6). **(J)** ImageJ pixel intensity quantification for the GFAP signal in the confocal images normalized to 1, demonstrating relative intensity of protein localization within the retina. **(K)** Overlay of *GFAP* RNA expression normalized to 1 and ImageJ protein localization normalized to one. The discordance in the gene expression and protein localization suggests that the first GFAP protein intensity peak at 24 hpl is due to a redistribution of existing GFAP protein and the second protein intensity peak at 5 dpl is due to increased transcription of *GFAP*. **(L)** Graph of transcript pseudo-counts for *PCNA* from 3′mRNA-seq of individual adult zebrafish retinas for each timepoint (*n* = 6). **(M)** ImageJ pixel intensity quantification for the PCNA signal in the confocal images normalized to 1, demonstrating relative intensity of protein localization within the retina. **(N)** Overlay of *PCNA* RNA expression normalized to 1 and ImageJ protein localization normalized to one. Scale bar represents 5 μm.

It is well established that zebrafish MG undergo a reactive gliosis response to photoreceptor damage and that they are the source of newly formed retinal progenitors (Thummel et al., 2008b; Nagashima et al., 2013). However, the timing of GFAP gene and protein expression during these events has not been fully clarified. To investigate these phenomena, we co-labeled the retinas with anti-GFAP and anti-PCNA antibodies to detect gliosis and cell-cycle re-entry, respectively, and then paired these findings with the transcript data for these genes (Figure 5). As previously reported (Thomas et al., 2016), we observed only very weak GFAP expression in MG basal endfeet processes in 0 h control retinas (Figure 5A). At 24 hpl, we observed that MG rapidly concentrated GFAP to their apical processes immediately adjacent to the photoreceptor nuclei (Figures 5B,C), but only exhibited a modest increase in *GFAP* gene expression at 24 hpl (Figure 5I). Surprisingly, we observed a strong peak in *GFAP* gene expression at 72 hpl (Figure 5I), during the peak of progenitor proliferation. Furthermore, this fourfold peak in gene expression was followed by a second peak in GFAP IHC positivity at 5 dpl (Figures 5E,J). Interestingly, we observed a different morphological presentation of the GFAP protein at this timepoint, distributed throughout the entire length of the MG apical-basal processes that spanned the retina (Figure 5E). Thus, this analysis highlights two phases of MG gliosis during retinal regeneration: an acute response that was focal to the retinal damage at the base of the ONL adjacent to the dying photoreceptors, and a secondary response at 5 dpl that had a distinct morphological distribution throughout the entire MG.

Of note, the dynamic gliotic response of the MG occurred concurrently to the initiation of a proliferative/regenerative response. At 36 hpl, we observed a continuation of GFAP concentration to the MG apical processes as MG reentered the cell-cycle, as visualized by the appearance of PCNA-positive MG cell bodies in the INL (Figure 5C). Clusters of migrating MGPCs were visualized by 72 hpl (Figure 5D), followed by a decrease in PCNA expression throughout the time course. The gene expression kinetics for *PCNA* closely matched what was observed via IHC, with the peak of gene expression occurring immediately prior to the peak in protein expression (Figures 5L–N).

### The Time Window Between Days 5 and 10 Post Phototoxic Lesion Represents a Period of Photoreceptor Differentiation

**FIGURE 6:**
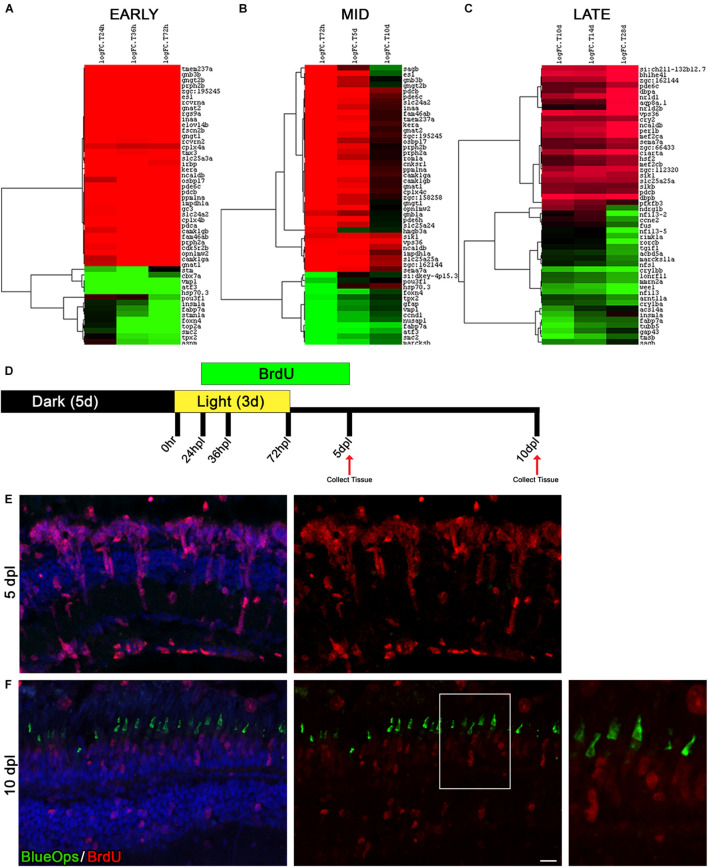
The time window between days 5 and 10 of regeneration represents a distinct turning point toward differentiation. **(A–C)** A time-course analysis was performed on each of the following three time periods; early response (24, 36, and 72 hpl), mid-regeneration (72 hpl, 5, and 10 dpl), and late-regeneration (10, 14, and 28 dpl). The top 50 genes were run through hierarchical clustering with complete linkage in Gene Cluster 3.0. Genes upregulated from the 0 h baseline are in green and downregulated from the 0 h baseline are in red. All top 50 genes had an FDR < 2 × 10^− 9^. **(D)** To trace newly generated cells during the process of regeneration, cells were labeled with BrdU added to the fish water from 24 hpl (prior to MG cell cycle entry) to 5 dpl (past the peak proliferation timepoint) and harvested at 5 and 10 dpl. **(E,F)** Retinal sections were co-labeled with anti-BrdU (red) and anti-Blue Opsin (green) at 5 dpl **(E)** and 10 dpl **(F)**. Nuclei were stained blue with TO-PRO-3. We did not observe immunolocalization of Blue Opsin at 5 dpl **(E)**. In contrast, the expansion in the bottom right highlights that the Blue Opsin signal seen in the new outer segments of cones at 10 dpL is coming from the BrdU-positive, newly derived cone photoreceptors as a result of regeneration. The heat map paired with the immunolabeling comparison of the 5–10 dpl timepoints demonstrates that the period of time between 5 and 10 dpl likely represents a major cell fate decision point in which stem cell pathways are being shut down and pro-differentiation pathways are being turned on. Scale bar represents 5 μm.

To investigate the dynamics of gene expression across the different phases of the regeneration process, we assembled heatmaps of the top 50 genes at each time point during three time windows: early response (24, 36, and 72 hpl), mid-regeneration (72 hpl, 5 and 10 dpl), and late regeneration/differentiation (10, 14, and 28 dpl) (Figures 6A–C). In the early response phase, gene expression was either significantly down or up-regulated (Figure 6A). In contrast, the late response/differentiation dataset showed changes in gene expression that were more varied (Figure 6C). Most intriguing, however, we observed a stark contrast in the mid-regeneration heatmap from 5 to 10 dpl (Figure 6B). We hypothesized that these transcriptional changes correlated with a switch from progenitor cell proliferation to photoreceptor differentiation, as was previously suggested using a different damage model (Raymond et al., 2006). To test this hypothesis in our model, we labeled dividing cells from 24 hpl through 5 dpl during the regeneration time-course by BrdU incorporation, which spanned the onset and peak of MGPC proliferation (Figure 6D). Eyes were collected for IHC analysis at 5 and 10 dpl and labeled with an anti-BrdU antibody (Figures 6E,F). In addition, we co-labeled these retinal sections with anti-Blue Opsin, as an indicator of photoreceptor differentiation. At 5 dpl, large columns of BrdU-positive progenitors were observed, with the majority of the BrdU-positive cells residing in the ONL (Figure 6E). There was no histological evidence of any differentiated blue cone photoreceptors at this time point (Figure 6E). At 10 dpl, BrdU immunolabeling was generally weaker, indicating dilution of the signal due to continued cell proliferation (Figure 6F). However, BrdU-positive blue cone photoreceptors were clearly visualized at this time point (Figure 6F), confirming that the time period between 5 and 10 dpl represents a window of photoreceptor differentiation in our transcriptomic dataset. Consistent with this, many of the genes that increased significantly in expression from 5 to 10 dpl were photoreceptor specific, including *phosphoducin (pdcb)*, *opn1mw2*, *sagb*, and *gngt2b* (Figure 6B). This dataset could serve as a source of future studies on genes of interest that control photoreceptor differentiation.

### The 28 dpl End-Point of Retinal Regeneration Exhibits a Transcriptome Distinct From the Naïve Retina

**FIGURE 7:**
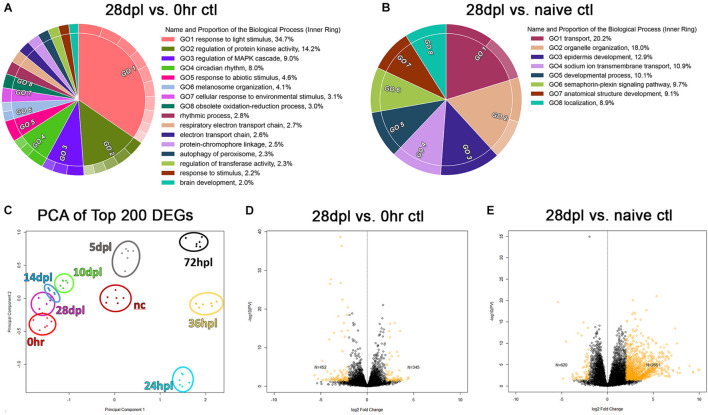
28 days post light does not represent transcriptional recovery to either the 0 h, or naïve control transcriptional baseline. **(A)** Gene Ontology (GO) was performed on the time-course analyses of all significantly differentially regulated genes (DEGs) (*p* < 0.002) comparing dark-adapted 0 h controls to 28 dpL retinas and **(B)** non-dark-adapted naïve controls to 28 dpL retinas. Significant DEGs were run through the “Reduce and Visualize Gene Ontology” (REVIGO) software to remove redundant GO terms based on similarity. Circular Visualization plots were generated using the Circular Gene Ontology terms Visualization (CirGO) algorithm displaying up to 20 of the most represented categories. Inner rings represent the hierarchical summary categories identified by the REVIGO software that contain the subcategories in the outer rings that fall under the umbrella term (not shown). Keys to the right of each graph represent labels for the inner ring categories. **(C)** Principle component analysis constructed using the top 200 genes differentially regulated as compared to the 0 h baseline, with the addition of the non-dark-adapted naïve control group (“nc” dark red) which clustered distinct from both the dark-adapted 0 h controls and the 28 dpL retinas. **(D,E)** Volcano plots highlighting the remaining significant DEGs in yellow (*p* < 0.05) at **(D)** 28 dpL compared to dark-adapted 0 h controls and **(E)** 28 dpL compared to naïve controls.

We hypothesized that the transcriptome of the regenerating retinas would incrementally return to the undamaged, dark-adapted 0 h control retinas, which was supported by our initial PCA analysis (Figure 1C). However, when we compared dark-adapted 0 h transcriptomes to the 28 dpl samples, we found 452 significantly downregulated DEGs and 345 significantly upregulated DEGs at 28 dpl (Figure 7D; *p* < 0.002 was used as a cutoff). Gene-ontology analysis of the DEGs highlighted several major gene categories that reflected an effect of the 5-day dark adaptation prior to the light treatment in our 0 h control group. For example, some of the top categories were “response to light stimulus,” “circadian rhythm,” and “response to environmental stimulus” (Figure 7A). Therefore, we repeated our analysis with non-dark adapted, age-matched naïve controls to determine whether the regenerated transcriptome returned to the naïve state. We found that there were overall fewer GO categories in the comparison of 28 dpl retinas to naïve controls than in the comparison of 28 dpl retinas to dark-adapted 0 h controls, and all of the visual processing related categories disappeared in this new analysis (Figure 7B). However, new GO categories appeared that suggest an unresolved transcriptome at 28 dpL (Figure 7B), and we observed more DEGs in the comparison of 28 dpl retinas to naïve controls than in the comparison of 28 dpl retinas to dark-adapted 0 h controls (Figures 7D,E).

Finally, we observed a distinct alteration in the transcriptional state due to the dark adaptation that is common to all light damage paradigms. This phenomenon was most visually apparent in the PCA plot of the top 200 DEGs in each dataset (Figure 7C). We noted that the naïve controls clustered tightly within their sample group, but spatially distinct from both the dark-adapted 0 h controls and the 28 dpl retinas (Figure 7C). Collectively, these findings suggest that—despite widely reported functional recovery—28 dpl retinas did not return to a naïve age-matched retinal transcriptome.

**FIGURE 8:**
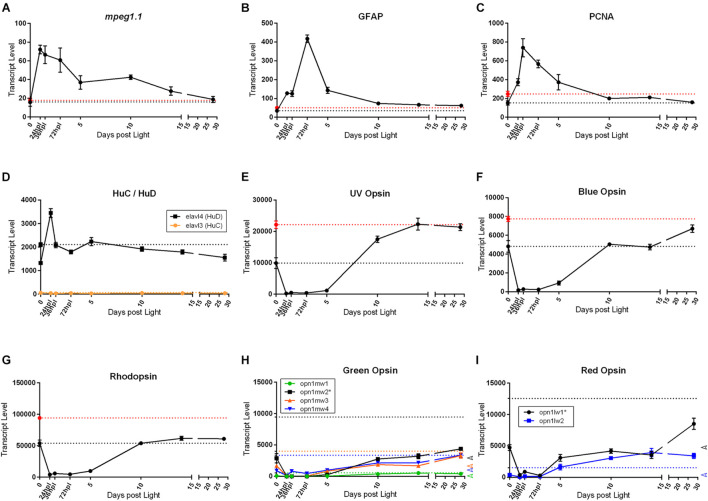
Common genes studied in retinal regeneration with dark adapted 0 h control, and naïve, non-dark adapted control baselines. All graphs in this figure represent transcript pseudo-counts for each of the genes listed above the plots from 3′mRNA-seq of individual adult zebrafish retinas for each timepoint (*n* = 6). In plots **(A–G)**, the black dotted line on the y-axis represents the baseline gene expression for the dark-adapted 0 h controls, the red dotted line represents the gene expression baseline of age-matched, non-dark-adapted naïve control retinas. In plots **(H,I)**, colored dotted lines on the y-axis correspond to the naïve control baseline gene expression corresponding to the same color of the genes in the key. The dark-adapted control baselines are not highlighted in these plots for ease of interpretation. Instead, on the far-right axis, empty arrowheads corresponding to the 0 h control baseline transcript values are displayed for reference. For inflammatory **(A)**, gliotic **(B)**, proliferative **(C)**, and inner nuclear layer **(D)** markers, we observe that the dark-adapted 0 h controls and the naïve controls have similar baseline expression and the expression pattern generally resolves to baseline at 28 dpl. The opsins have several distinct patterns of recovery. **(E,F)** Short wavelength cone opsins recover to the naïve control gene expression baseline whereas **(G)** Rod photoreceptor opsin expression returns to the dark-adapted 0 h control baseline. **(H,I)** Medium and long wavelength opsins appear to have different patterns of recovery even between paralogs of the same opsin, with some paralogs returning to the naïve control baseline and some returning to the dark-adapted 0 h control baseline. Asterisks in paralog keys in **(H,I)** represent the most dominantly expressed paralog at the 0 h baseline for opsins with multiple paralogs.

As a final exploration of the transcriptional differences between the naïve control, 0 h dark-adapted control, and 28 dpl retinas, we re-visited our original investigations into individual genes highly studied in the retinal regeneration literature. These included inflammatory markers (Figures 4, 5), stem cell genes (Figure 5), and the opsins (Figures 2–4). We normalized and quantified the transcript numbers of the newly added naïve control transcriptomes and plotted them as a new baseline on the gene expression graphs (Figure 8). We noticed three distinct phenomena in the patterns of gene expression recovery at 28 dpl compared to naïve control retinas: (1) genes that did not change drastically in expression from dark adaptation and returned to a similar baseline as naïve controls at 28 dpl, (2) genes that did change due to dark adaptation, but recovered to the naïve retina transcriptional baseline, and (3) genes that did change due to dark adaptation and recovered to the dark-adapted 0 h baseline. The first category included the inflammatory and gliosis markers *mpeg1.1* (Figure 8A) and *GFAP* (Figure 8B), both of which appeared relatively unaffected in expression by the dark adaptation and recovered back to pre-damage levels. In this same category, we also have the cell cycle marker *PCNA* (Figure 8C) and the amacrine/ganglion cell markers HuC and HuD (*elavl3* and *elavl4*, respectively) (Figure 8D). Interestingly, the opsin genes followed different recovery patterns based on type of opsin. Not surprisingly, all opsins significantly decreased during the 5-day dark adaptation, but the short wavelength cone opsins [UV (opn1sw1) and Blue (opn1sw2)] recovered completely to the naïve control baseline of gene expression (Figures 8E,F). In contrast, *rhodopsin* expression never reached the naïve control baseline, but instead, recovered to the dark-adapted baseline (Figure 8G). Lastly, the medium and long wave length opsins [Green Opsin (*opn1mw1-4*) and Red Opsin (*opn1lw1-2*)] have multiple paralogs, which also exhibited distinct recovery patterns (Figures 8H,I). While the most dominantly expressed Green Opsin at the dark-adapted 0 h baseline did not recover to the naïve control baseline transcription level, all non-dominant paralogs (*opn1mw2-4*) appeared to recover close to the naïve control baseline (Figure 8H). Red Opsin appeared to follow a similar pattern, though in this case, the non-dominantly expressed paralog (*opn1lw2*) recovered to a level higher than both the naïve and 0 h control baselines (Figure 8I). To our knowledge, this represents the first investigation into the differential transcriptional recovery profiles of the opsin genes upon phototoxic lesion.

## Materials and Methods

### Fish Maintenance

Adult (6–9 months) *albino* (*alb*) zebrafish were used for all experiments. Unless otherwise noted, fish were maintained under a daily light cycle of 14 h light (250 lux):10 h dark at 28.5°C (Westerfield, 1995) and fed a combination of flake food and brine shrimp. The Institutional Animal Care and Use Committee at Wayne State University approved all procedures used in this study (Protocol # 19-02-0970).

### Intense Light Exposure and Tissue Collection

A photolytic damage model was used to destroy rod and cone photoreceptors (Thomas et al., 2012). First, 24 adult *albino* fish per treatment group were dark adapted for 5 days. Next, the 0 h control group tissue was harvested and the remaining fish were subjected to a two-part photolytic damage for three days as previously described (Turkalj et al., 2021), and then transferred back to standard light:dark conditions. Briefly, fish were subjected to 30 min of intense ∼100,000 lux broad spectrum white light exposure from a mercury halide bulb through a liquid light guide (Leica Microsystems, Cat#EL6000, Feasterville, PA). Fish were then immediately transferred to a secondary continuous light exposure of lower intensity (∼10,000 lux) utilizing four 250 W halogen bulbs (Woods Home Products, cat#L21, Carrollton, Georgia). Eyes were harvested at the following time points post-onset of light treatment: 24 h post light (hpl), 36, 72 hpl, 5 days post light (dpl), 10, 14, and 28 dpl (Figures 1A,B). The full experiment was performed in duplicate with *n* = 3 animals, resulting in *n* = 6 animals per condition in total. Prior to tissue harvesting, animals were euthanized in a 1:500 dilution of 2-Phenoxyethanol (Millipore Sigma cat#77699, St. Louis, MO) according to the approved protocol.

### BrdU Incorporation

For the BrdU study, 10 adult (6–9months) *albino* fish were subjected to the same light treatment procedure described above, except that at 24 hpl, fish were transferred to a 1 L solution containing 0.66 g of NaCl, 0.1 g Neutral Regulator (SeaChem Laboratories, Inc. Stone Mountain, GA), and 1.5 g (5 mM) BrdU (Sigma, Saint Louis, MO). After 48 h in the BrdU solution, fish were transferred into a fresh preparation of the BrdU solution. After an additional 48 h of BrdU incorporation (i.e., at 5 days post onset of light treatment), fish were placed back into fresh system water and split into two groups. 5 of the animals were sacrificed and their eyes were harvested for immunohistochemistry; the remaining 5 were returned to normal conditions for an additional 5 days, at which point they were euthanized, and their eyes were harvested for immunohistochemistry at 10 dpl.

### Immunohistochemistry

Two different immunohistochemistry protocols were used for the primary antibody incubation. Standard immunohistochemistry was performed exactly as previously described (Thummel et al., 2008a) using the following primary antibodies: rabbit anti-Rhodopsin antisera (gift from David Hyde; 1:5,000), mouse anti-PCNA (Sigma; 1:1,000), rabbit anti-Blue Opsin (gift from David Hyde; 1:500), rabbit anti-UV Opsin (gift from David Hyde; 1:1,000), rabbit anti-Red Opsin (gift from David Hyde; 1:500), rabbit anti-Green Opsin (gift from David Hyde; 1:500), rabbit anti-GFAP (DakoCytomation; 1:500), mouse anti-4C4 (gift from Peter Hitchcock; 1:250), mouse anti-HuC/D (Invitrogen; 1:50). For the use of rat anti-BrdU (Accurate Chemical; 1:200), an antigen retrieval procedure was used exactly as previously described (Thummel et al., 2008a). For both primary antibody procedures, standard secondary antibody procedures were followed as previously described (Thummel et al., 2008a) using AlexaFluor-conjugated 488 and 594 anti-primary (1:500, Life Technologies, Grand Island, NY) and a nuclear stain (TO-PRO-3 “TP3”; 1:750; Life Technologies, Grand Island, NY). Slides were covered with a coverslip using ProLong Gold mounting medium (Molecular Probes, Eugene, OR).

### Confocal Microscopy and Image Quantification

Confocal microscopy was performed using a Leica TCS SP8 confocal microscope. Images were taken in a single plane with the same exposure settings over a 300-micron linear distance on the central dorsal retina. The central dorsal retina was chosen for analysis because (1) it has been well characterized by the field that this region is most significantly damaged by the light damage paradigm, thus leading to the most robust regenerative response and (2) using the same region for all IHC analysis eliminated regional differences observed in photoreceptor loss induced by phototoxic lesions (Thomas et al., 2012). Quantification of detected antigens was performed using ImageJ to quantify mean fluorescence intensity of a single fluorescence channel for each antibody used. Regions of interest were drawn with identical dimensions to include the full retina thickness and the entire 300-micron width of the images. Images were directly quantified and normalized to the 0 h baseline within single imaging session to avoid confounding microscope and staining variability. For the TUNEL assay, images were acquired exactly as described above, but positive nuclei were counted by hand in each image. Statistical differences between groups was analyzed by a one-way ANOVA followed by a *post-hoc* Tukey test using a *p* < 0.05 as a cut-off.

### Retinal Dissection and RNA Isolation

Left eyes from individual animals were harvested at the aforementioned time points and retinal tissue was isolated from the lens and sclera using the following procedure (Figures 1A,B). First, the eye was placed on a sterile Petri dish with the optic nerve facing up. Next, the optic nerve was transected at the back of the eye, leaving a small hole. Size 35 surgical scissors were inserted vertically into the hole and a cut was made in the sclera, radiating from the hole. The eye was then pinched with forceps on the opposite side of the cut and the retina, vitreous, and lens were extruded onto a sterile Petri dish. Using forceps, the lens was then carefully separated from the retina and the retina tissue was transferred into empty, pre-chilled, nuclease-free Eppendorf tubes. RNA was immediately isolated using the Direct-zol^TM^ RNA MicroPrep kit (Zymo Research, Irvine CA), according to the manufacturer protocol.

### cDNA Library Preparation and 3′mRNA-Seq and Analysis

Purified RNA was submitted to the Genome Sciences Core at Wayne State University for RNA quality control, cDNA library preparation, and sequencing. In brief, sequencing libraries were generated from 250 ng of total RNA using Lexogen’s Quantseq 3′mRNA-Seq Library Prep Kit FWD before sequencing on a NovaSeq (minimum of 5 M reads per sample). Reads were aligned to the zebrafish genome (Build dR10) (Dobin et al., 2012) and tabulated for each gene region (Anders et al., 2014). Differential gene expression analysis was used to compare transcriptome changes between conditions (6 replicates per condition across two separate experiments) (Robinson et al., 2009). In addition, separate time series analyses to identify genes involved in the early, mid and late stage changes were also run. Significantly altered genes (|log fold change| = 2; *p*-value = 0.05) were used to identify affected pathways (Huang et al., 2009). Mapped reads and pairwise comparisons for the entire dataset can be found in Supplementary Table 1. The datasets presented in this study can be found in the NCBI Gene Expression Omnibus, accession no: GSE180518.

## Discussion

There are two general approaches used to study zebrafish retinal regeneration: targeted genetic studies of individual pathways, and large, unbiased traditional RNA sequencing studies. Weaknesses of the latter is high cost and the requirement of large amounts of starting material, which collectively often limit the scope these experiments. With this study, we utilized each biological replicate animal for both morphological analysis via IHC, and transcriptomic studies via 3′mRNA-seq. In doing so, we take advantage an underutilized and relatively inexpensive modern technique for transcriptomic analysis and pair it with quantification of IHC and morphological analysis. Together, we provide an updated expression map of the complete 28-day cycle of retinal degeneration and regeneration in adult zebrafish. In addition, we also show several novel findings not previously reported in this well-characterized regeneration model that warrant future investigations. Finally, we also tested the hypothesis that the 28-day endpoint of retinal regeneration represented true transcriptional recovery of the retina to an undamaged state. We provide evidence that it does not, which may challenge how we define complete regeneration.

There are both advantages and disadvantages for using the 3′mRNA-seq method for transcriptional profiling. Because there is no fragmentation of the RNA with 3′mRNA-seq, the number of reads per transcript directly reflects the number of transcripts present in the cell. In contrast, it has been reported that traditional RNA-seq presents a bias toward longer transcripts because they are fragmented into more pieces, and thus have more reads (Stark et al., 2019). Therefore, 3′mRNA-seq, could be thought of as a non-gene specific whole-transcriptome Taqman assay. One example of how 3′mRNA-seq offers information on relative expression levels is that we report for the first time the comparative levels of the different opsin genes at a 5-day dark-adapted baseline prior to damage to a non-dark-adapted naïve control, revealing the profound effect that dark-adaptation has on the quantity of transcripts present for each opsin. We also report which Red and Green Opsin paralogs are expressed in the adult retina. The one disadvantage that 3′mRNA-seq presents is the lack of information on transcript variants. Because 3′mRNA-seq only captures the 3′end of the gene, no information on splice variants can be obtained from these data sets, only gene expression levels. Because of the cost-effectiveness, simplicity of data analysis, and low starting material requirement, several groups have compared the data reliability of 3′mRNA-seq to traditional RNA sequencing with promising conclusions (Ma et al., 2019). The gene expression patterns in well-established models are highly replicated between the two sequencing methods with minor differences. RNA-seq tends to capture 10–20% more differentially expressed genes than 3′mRNA-seq, and RNA-seq tends to be biased toward long transcripts, whereas 3′mRNA-seq captures more short transcripts (Corley et al., 2019; Ma et al., 2019). Depending on the experimental question, 3′mRNA-seq can provide access to reliable whole transcriptome gene expression information for lower cost, less downstream data processing, and less starting material.

Based on previous studies, we chose a 28-day regeneration period with 8 time points for morphological and transcriptomic analysis. The time points (0 h, 24, 36, 72 hpl, 5, 10, 14, and 28 dpl) were chosen to investigate early, middle, and late regenerative stages. Five time points were concentrated in the first week of regeneration because it is well-established that these time points cover the most dynamic cellular processes in the regenerating zebrafish retina (Vihtelic and Hyde, 2000; Kassen et al., 2007; Thummel et al., 2008a; Thomas et al., 2012; Lenkowski and Raymond, 2014). Individual transcriptomes from single retinas as biological replicates were sequenced with the 3′mRNA-seq method, and, as demonstrated by the PCA analysis in Figure 1C, the biological replicates clustered distinctly, and tightly, highlighting the accuracy and precision this method provides, which allowed us confidence in the reliability of these data.

Because the phototoxic lesion protocol used in these studies targets the photoreceptors in the outer retina, we wanted to examine the dynamic morphological and genetic changes associated with photoreceptor degeneration/regeneration over the entire 28-day period. As can be appreciated in Figures 2–4, we highlighted the rapid destruction of cone and rod photoreceptors, noting by IHC analysis, destruction already present at 24 hpl and absence of intact cones and rods by 5 dpl. When using ImageJ to quantify protein localization as a proxy for protein level, the IHC imaging suggested that following light damage there is an observed hypertrophy of the outer retina as the photoreceptors are destroyed and dying cells are compartmentalized and broken down. Ironically, the compaction of photoreceptor debris increased fluorescence pixel density, resulting in an artificial increase in protein localization in ImageJ. This is an important caveat to using this unbiased quantification method.

When comparing the cone and rod IHC data to the gene expression data presented in Figures 2–4, it is clear that there is an inverse relationship between the transcription profiles and the apparent protein quantifications at the early time points from 0 to 72 hpl. The gene expression data of *rhodopsin* and all cone opsins showed a rapid drop in transcript levels from 0 h to 72 hpl, with gene expression beginning its upward trajectory between 72 hpl and 5 dpl. This upward trajectory of gene expression recovery generally preceded evidence of opsin protein by IHC at 5 dpl, suggesting that the opsin genes may be expressed in photoreceptor precursors, or newly made photoreceptors, prior to their terminal differentiation. The exception to this observation was Red Opsin, where we observed very weak expression of Red Opsin at 5 dpl (Figure 2P). This observation may reveal that the differentiation of cones is kinetically distinct for each cone type, and potentially even between opsins within the same cone, as Red and Green Opsins are housed within the same double-cone in zebrafish (Branchek and Bremiller, 1984; Endeman et al., 2013).

An interesting observation of gene paralog expression arose when we labeled amacrine and ganglion cells with the HuC/D antibody to confirm that the inner retina remained undamaged in our paradigm. While the HuC/D antibody co-labels the two proteins HuC and HuD, making them indistinguishable, with the transcriptome data we were able to investigate these two genes, *elavl3* and *elavl4*, separately. As can be seen in Supplementary Figure 2, the HuC/D+ neurons remained morphologically intact and evenly distributed. There was a slight increase in MFI from 36 to 72 hpl, but no change in morphology (Supplementary Figures 2C,D,J). Interestingly, *elavl3* (HuC), was not expressed highly in the retina. *Elavl4 (*HuD*)*, however, exhibited over a twofold increase in gene expression immediately following injury, corresponding to the slight increase in MFI at 36–72 hpl (Supplementary Figure 2I). It is possible that the small increase in MFI at these timepoints could be a result of this increase in *elavl4* (HuD) gene expression, as it was the dominant transcript in the adult retina in this experiment, which has not been previously reported. It is currently unknown how this response may play a role in the regenerative process, but it is possible that because the MG span the entirety of the retina, they could transport damage signals from the outer retina to the inner retina, resulting in transcriptome changes in undamaged inner nuclear neurons. It is also possible that the loss of normal photoreceptor transduction could have an indirect effect on the transcription of down-stream/third-order inner neurons.

As the role of inflammatory cells in the regenerating retina is an area of increasing interest and investigation (Mitchell et al., 2018; Bollaerts et al., 2019; Silva et al., 2020; Zhang et al., 2020), we aimed to map the timeline of DNA damage and inflammation response in our model. 4C4+ microglia reside primarily in the inner and outer plexiform layers in undamaged retinas and migrate to the area of damage upon insult (Raymond et al., 2006; Craig et al., 2008). To assess DNA damage, a TUNEL assay was performed and the results corresponded closely with the presence of 4C4+ microglia and degenerating photoreceptors shown in Figure 4. First, at 24–36 hpl, the rods underwent a visible hypertrophy (Figures 4B,C), which correlated with the highest TUNEL positivity in the ONL at these time points (Supplementary Figures 1B,C). This sequence of events is consistent with photoreceptor damage and debris providing signals for inflammatory cell migration and activation (Nelson et al., 2013; Nagashima et al., 2019). While the majority of morphologically active microglia are no longer present at the 28 dpl time point, interestingly, we see the re-emergence of a few non-active 4C4 positive cells in the INL of the retina (Figure 4S). It is possible that these are sentinel cells, residing permanently to be ready to remove debris from future insults, as suggested by a repeated phototoxic lesion model previously described by our group (Ranski et al., 2018). An alternative possibility is that a residual chronic level of inflammation remains at 28 dpl and never returns to baseline.

The response of the MG to phototoxic lesion has been well characterized for several years. To validate both our lesion protocol, and our readout methods, it was necessary to label and plot out transcriptome data for two classic genes involved in zebrafish retinal regeneration, *GFAP* and *PCNA.* These genes represent the dynamic and tightly regulated balance between gliosis and proliferation/regeneration, respectively (Thummel et al., 2008a, b; Thomas et al., 2016). At 24 hpl, GFAP was morphologically concentrated at the outer appendages of the MG, which resulted in the first peak of protein quantification. This initial increase in GFAP detection by IHC corresponded to only a modest increase in *GFAP* gene expression (Figure 5L) at 24 hpl. Since we did not have a tissue collection timepoint between 0 and 24 hpl, it is possible that a very early increase in *GFAP* gene expression accounted for the increased GFAP protein expression in the MG appendages in the ONL. Alternatively, the initial increase in IHC detection could be attributed to an intracellular kinetic response of the MG, redistributing this intermediate filament of the cytoskeleton to the site of injury at the ONL. It is also possible that a combination of these two possibilities occurs. Regardless of the mechanism, it should be noted that GFAP expression initially localizes to the area of acute damage in multiple damage models in rodents and zebrafish (Bringmann and Wiedemann, 2012; Krigel et al., 2016; Thomas et al., 2016), which is consistent with our finding. The second peak of GFAP IHC positivity occurred at 5 dpl and was morphologically distributed throughout the entire length of the MG from ONL to INL (Figures 5E,M). This increase in GFAP positivity was likely due to the fourfold increase in *GFAP* gene expression at 72 hpl (Figure 5L). This is a novel finding and maybe akin to GFAP expression being more globally distributed throughout the MG in chronic damage models of retinal detachment and blue-wave light exposure (Bringmann and Wiedemann, 2012; Krigel et al., 2016).

Because zebrafish MG are dynamically regulated, they are able to concurrently exhibit acute and chronic gliotic phenotypes along with a proliferative stem cell response, all the while maintaining retinal homeostasis. It was previously described that at the 36 hpl time point, reactive gliosis occurs concurrently with a robust upregulation of *PCNA* (Thomas et al., 2016), however, the persistence of the gliotic response of the MG beyond the 36 hpl time point has not been reported in the zebrafish phototoxic lesion paradigm. The data presented in Figure 5 suggest a much more dynamic orchestration of multiple processes at once and potentially two distinct gliosis phenotypes, an early gliotic and neuroprotective response that does not correlate with a robust increase in *GFAP* gene expression, and a later, uncharacterized global response subsequent to a large increase in gene expression. Importantly, our approach to directly compare morphology, protein localization quantification, and gene expression changes at each time point allowed us to visualize these data in a new way and highlight new observations.

The next experiment performed in this investigation highlighted an important window of differentiation in the zebrafish retina that warrants future investigation. Through careful observation of the PCA plot (Figure 1C) and the heat maps generated in Figure 6, we noticed that a large shift appeared in the transcriptome profiles between days 5 and 10 post lesion. To determine if differentiated opsins were present in newly generated photoreceptor precursors, we labeled new MGPCs with BrdU in live animals, harvested the tissue at 5 and 10 dpl, and co-labeled with antibodies targeting BrdU and Blue Opsin. As demonstrated in Figure 6E, there is no evidence of Blue Opsin protein at 5 dpl, but at 10 dpl, Blue Opsin is detectable and cone morphology is obviously apparent (Figure 6F). This indicates that our 5 and 10 dpl datasets can be used for future investigations to probe for important differentiation factors in the final stages of MGPC fate determination and maturation.

Lastly, upon investigation into the transcriptional recovery of the regenerating retina at the 28-day endpoint established by the field, we were first surprised by the degree to which *rhodopsin* and the cone opsins all overcompensated their gene expression above the 0 h pre-damage baseline (Figures 2I,T, 3I,T, 4I). We initially hypothesized that this overcompensation indicated that the photoreceptors were still be in the process of maturation. However, when we next investigated the GO analysis of the significant DEGs remaining at 28 dpl, we realized that the highlighted categories pointed to changes in visual stimuli and patterning the circadian clock (Figure 7A), indicating significant transcriptional alteration due to the 5-day dark adaptation. We therefore repeated our analysis using non-dark-adapted, age-matched naïve controls that were in normal light:dark conditions. We hypothesized that these new controls would reveal that the 28 dpl transcriptome returned to a near normal naïve state.

However, we observed several interesting results that open further questions into the transcriptional recovery of the retina. First, there were overall fewer GO categories in the naïve control comparison to 28 dpl retinas than in the dark-adapted 0 h controls to the 28 dpl retinas, and the visual processing categories such as “response to light stimulus” and “circadian rhythm,” disappeared. Second, new GO categories appeared that suggest a transcriptome that remains unresolved at 28 dpL, for example “epidermis development” and “developmental processes.” Many of the genes in these categories seem to be involved in proteolysis and developmental patterning, including several proteolytic enzymes and *hox* genes. It is difficult to hypothesize why these pathways may be dysregulated still at 28 dpl. Despite apparent morphological recovery of individual photoreceptors as observed by IHC, Nagashima et al. (2013) demonstrated by wholemount analysis that the photoreceptor mosaic of the regenerated retina is perturbed compared to the naïve retina. Therefore, one possibility why genes in these GO categories may be dysregulated at this time point is an alteration or ongoing changes to the photoreceptor connectome. Alternatively, it is also possible that some of these genes could come from vitreal composition alteration, as several of the ECM proteins targeted in these categories are present in the vitreous (some vitreous material is included in the retinal harvest for sequencing as it is difficult to separate from the retinas). The final interesting finding from this investigation was the changes in gene expression due to the dark adaptation step that is common to all phototoxic lesions in this model (Figure 7C). Together, these findings clearly show that 28 dpl retinas are not transcriptionally recovered, despite functional recovery.

Finally, we re-visited the apparent overcompensation of opsin expression at 28 dpl in order to overlay the new naïve baseline control data onto the original graphs (Figure 8). With these new data, we noticed interesting trends within the opsin genes that have not been reported to date. The short wavelength cone opsins, UV and Blue Opsins, seem to have retained a transcriptional memory of the pre-dark adaptation baseline level as it is clear that the 28 dpl endpoint retinas regained full transcriptional recovery to the naïve control baseline (Figures 8E,F). In contrast, the opposite appears to be true of *rhodopsin*, which appears to have reached a steady plateaued transcriptional recovery reflective of the 0 h dark-adapted baseline. Lastly, the medium and long wavelength opsins, Green and Red Opsin, both of which have multiple paralogs, have different recovery trends even between paralogs. For example, the most highly expressed Green Opsin in the retina at baseline, *opn1mw2*, does not come close to recovering to the naïve retinal baseline expression of this paralog at 28 dpl, and appears to still be on an upward trajectory of gene expression, opening the question of whether or not with more recovery time this expression will reach the naïve retinal expression state. The remaining three paralogs of Green Opsin, however, appear to have fully recovered to the naïve retinal expression of these paralogs. Finally, the more highly expressed Red Opsin in the adult retina, *opn1lw1*, also did not return to a naïve retinal transcriptional state, but the lesser expressed paralog of this opsin, *opnl1w2*, surpassed the naïve transcriptional level. These gene expression patterns may suggest differential epigenetic programs of transcriptional level that may be influenced by dark adaptation. Furthermore, the apparent stabilization of the *rhodopsin* gene expression level to a dark-adapted transcriptional state may indicate a potential influence of the MG independent pathway of regenerating rods through existing rod precursor cells. In summary, these different patterns in gene expression recovery of the opsins open up new questions about what is considered the end-point of adult zebrafish retinal regeneration.

With the methods and analysis performed in this manuscript, we present a robust and economical way of investigating the dynamic process of zebrafish retinal regeneration in an unbiased way to allow for efficient comparative analysis. When pairing these transcript data with morphological analysis, we provide the field with an updated histological and transcriptomic map of zebrafish retinal regeneration and a transcriptome database which both affirms past studies, and provides new observations for future investigation. Furthermore, we reveal that the established 28-day functional recovery endpoint of retinal regeneration does not represent transcriptional recovery of the retina when compared to two separate baseline controls. By utilizing both dark-adapted and naïve retinal controls, we were able to discover nuances in the transcriptional recovery of the retina that pose new questions as to how we define complete retinal regeneration in the phototoxic lesion paradigm.

## Data Availability Statement

The datasets presented in this study can be found in online repositories. The names of the repository/repositories and accession number(s) can be found below: NCBI Gene Expression Omnibus, accession no: GSE180518.

## Ethics Statement

The animal study was reviewed and approved by the Institutional Animal Care and Use Committee—Wayne State University.

## Author Contributions

AK designed, performed all experiments, and wrote the manuscript. KG performed all bioinformatic analysis and assisted in generation of the PCA plot in Figure 1 as well as editing of the manuscript. RT designed the experiments and edited the manuscript. All authors contributed to the article and approved the submitted version.

## Conflict of Interest

The authors declare that the research was conducted in the absence of any commercial or financial relationships that could be construed as a potential conflict of interest.

## Publisher’s Note

All claims expressed in this article are solely those of the authors and do not necessarily represent those of their affiliated organizations, or those of the publisher, the editors and the reviewers. Any product that may be evaluated in this article, or claim that may be made by its manufacturer, is not guaranteed or endorsed by the publisher.
