# Supplementary material for: A Comparative Analysis of Gene and Protein Expression Throughout a Full 28-Day Retinal Regeneration Time-Course in Adult Zebrafish
Source: Front Cell Dev Biol. 2021 Nov 1;9:741514. doi: 10.3389/fcell.2021.741514 (PMC8591265; doi:10.3389/fcell.2021.741514)
Supplement: Supplementary file 1 [file Data_Sheet_1.DOCX]

**SUPPLEMENTAL METHODS**

**TUNEL**

A terminal deoxynucleotidyl transferase dUTP nick end labelling (TUNEL) assay was performed to detect DNA damage as previously described [1] using the ApoAlert™ DNA Fragmentation Assay Kit (TaKaRa, Kyoto, Japan) and biotinylated dNTPs (New England Biolabs, Ipswich, MD). AlexaFluor 488-conjugated Streptavidin (1:200, Invitrogen, Carlsbad, CA) was used for detection of incorporated biotinylated dNTPs and the slides were mounted and cover-slipped with Prolong Gold prior to imaging.

**SUPPLEMENTAL FIGURES**

**
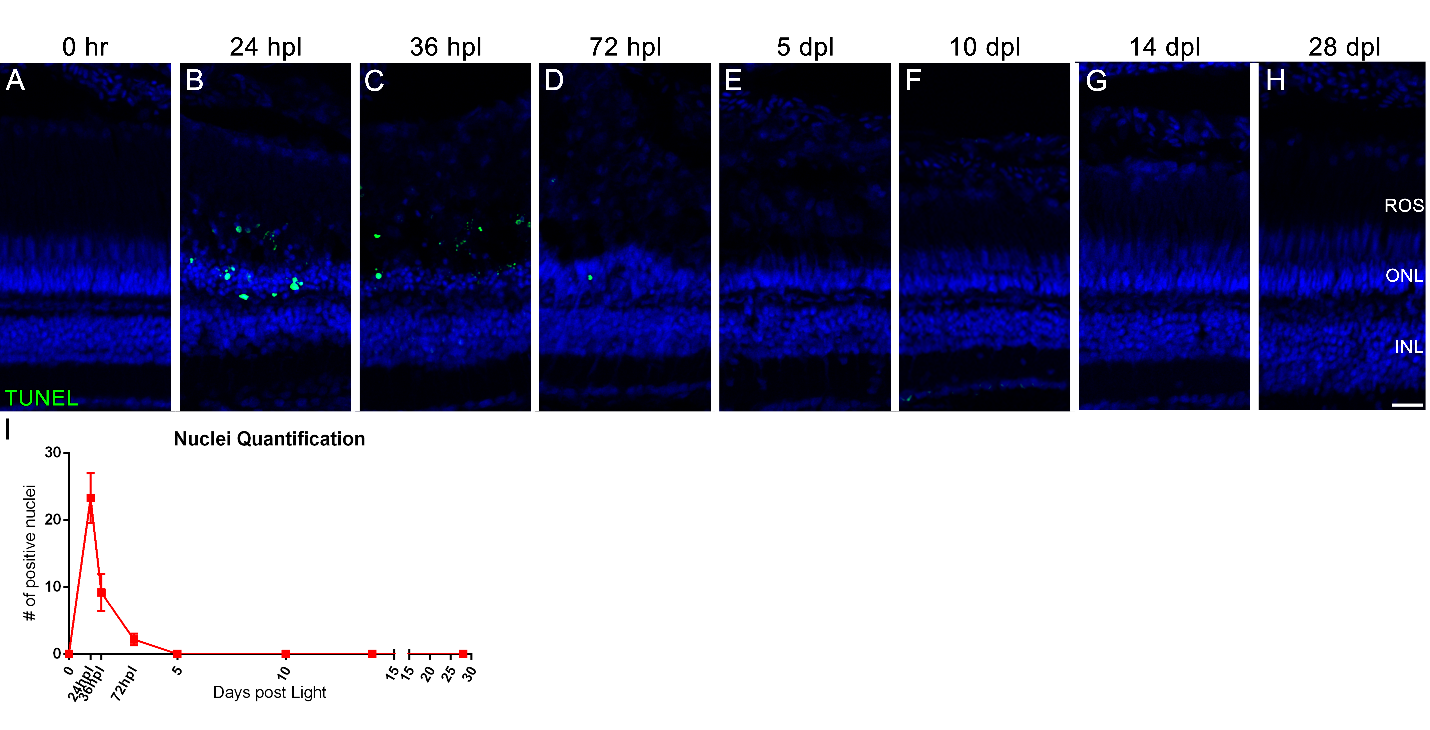
**

**Supplemental Figure 1. DNA damage peaks at 24-36hpl assessed by TUNEL assay.** A-H) DNA damage is demonstrated by TUNEL assay in these retinal sections collected at baseline (0hr) through 28 days post phototoxic lesion (dpl), hours post light are denoted (hpl). A TUNEL assay was performed on all sections and labeled with a streptavidin-AF488 conjugated secondary antibody (green) and nuclei were stained blue with TO-PRO-3(blue). TUNEL positivity peaks at 24hpl and disappears by 72hpl. (I) Graph of hand-counted positive TUNEL nuclei in images of retinas spanning about ~300µm linear distance (n=6). Scale bar represents 5µm.


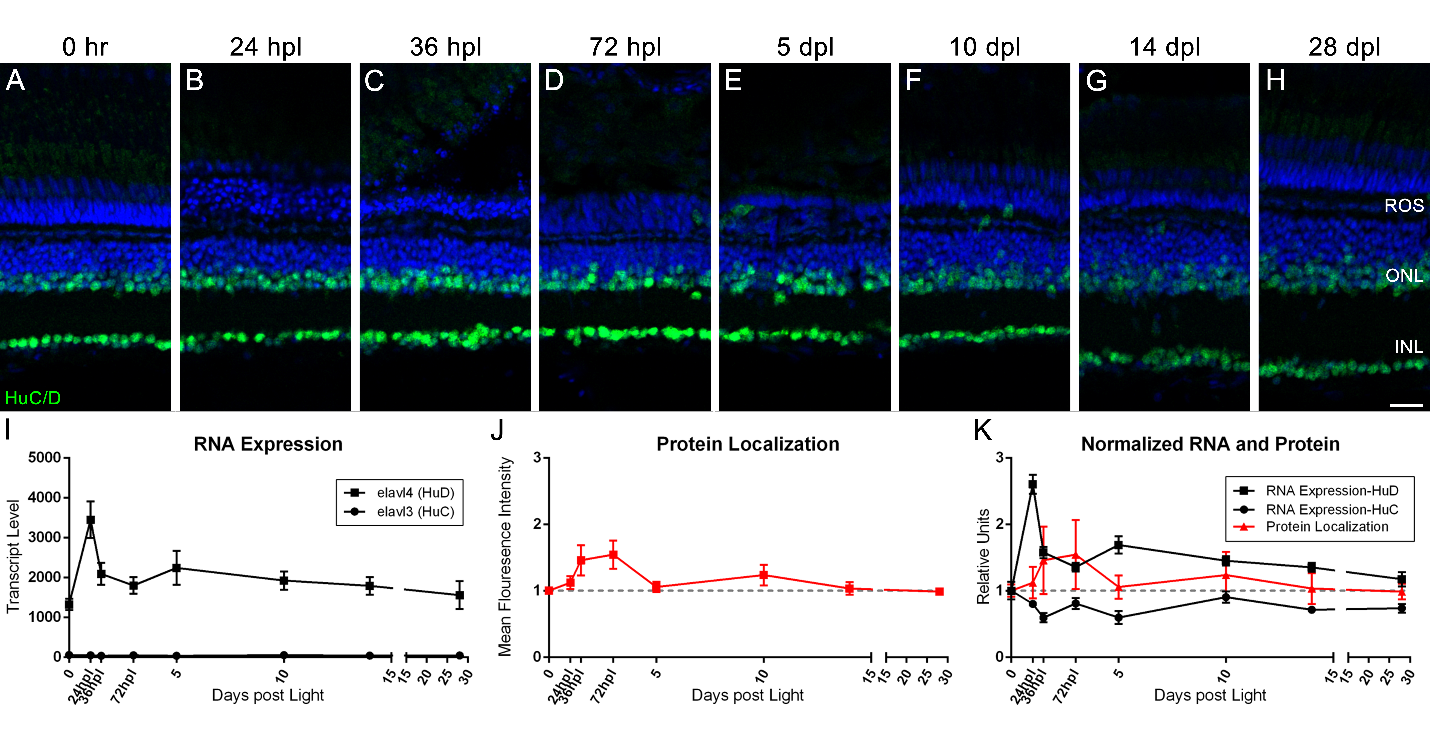


**Supplemental Figure 2. The inner nuclear layer remains intact with the phototoxic lesion protocol.** (A-H) Amacrine and ganglion cells located in the inner retina are relatively unaffected by the phototoxic lesion as demonstrated in these retinal sections collected at baseline (0hr) through 28 days post phototoxic lesion (dpl), hours post light are denoted (hpl). Sections are immunolabelled with HuC/D which is an antibody that stains for both of those antigens present on amacrine and ganglion _cells_. Nuclei were stained blue with TO-PRO-3 (n=5-6). (I) Graph of transcript pseudo-counts for *HuC* (*elavl3*) and *HuD* (*elavl4*) from 3’mRNA-seq of individual adult zebrafish retinas for each timepoint (n=6). *HuC* is expressed at much lower levels in the retina compared to *HuD*. (J) ImageJ pixel intensity quantification for the HuC/D signal in the confocal images normalized to 1, demonstrating relative intensity of protein localization within the retina. (K) Overlay of *HuC* and *HuD* RNA expression normalized to 1 and ImageJ protein localization normalized to one. This overlay indicates that the antigen likely to be representative of the genes expressed in these cell types in retinal tissue is likely the HuD­ component of the antibody. Scale bar represents 5µm.


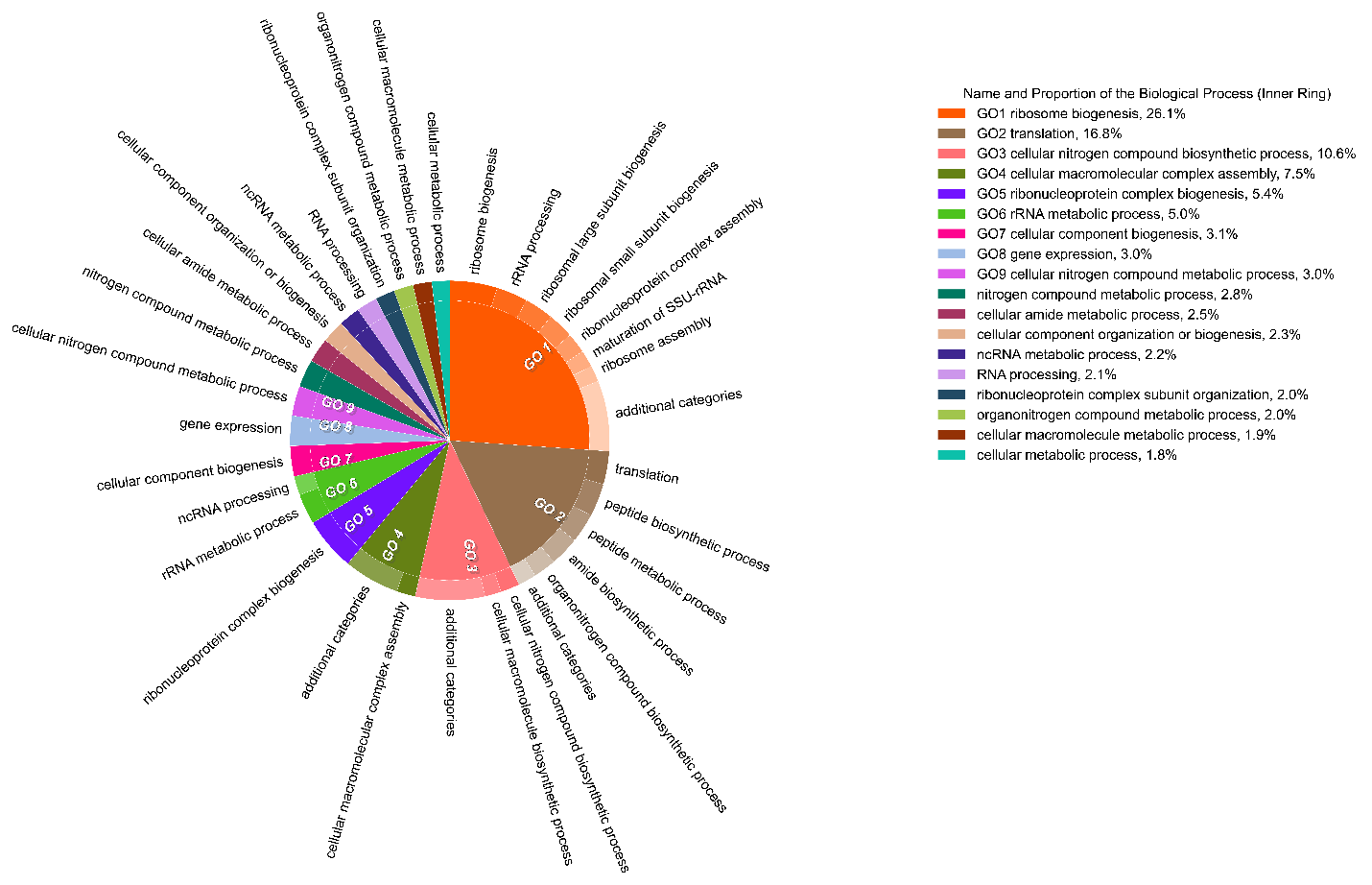


**Supplemental Figure 3. Gene Ontology analysis of early regeneration timepoints.** Gene Ontology (GO) was performed on the time-course analyses performed on the early response (24, 36 and 72hpl) subgroup of the eight original time points. All 350 GO terms with p<0.05 were run through the “Reduce and Visualize Gene Ontology” (REVIGO) software to remove redundant GO terms based on similarity. Circular Visualization plots were generated using the Circular Gene Ontology terms Visualisation (CirGO) algorithm displaying up to 20 of the most represented categories. Inner rings represent the hierarchical summary categories identified by the REVIGO software that contain the subcategories labelled in the outer rings. Keys below each graph represent labels for the inner ring categories. Subcategories within each inner-ring hierarchy are displayed as text in the outer ring. The early response CirGO plot is weighted heavily towards biochemical processes such as transcription and translation machinery.

**
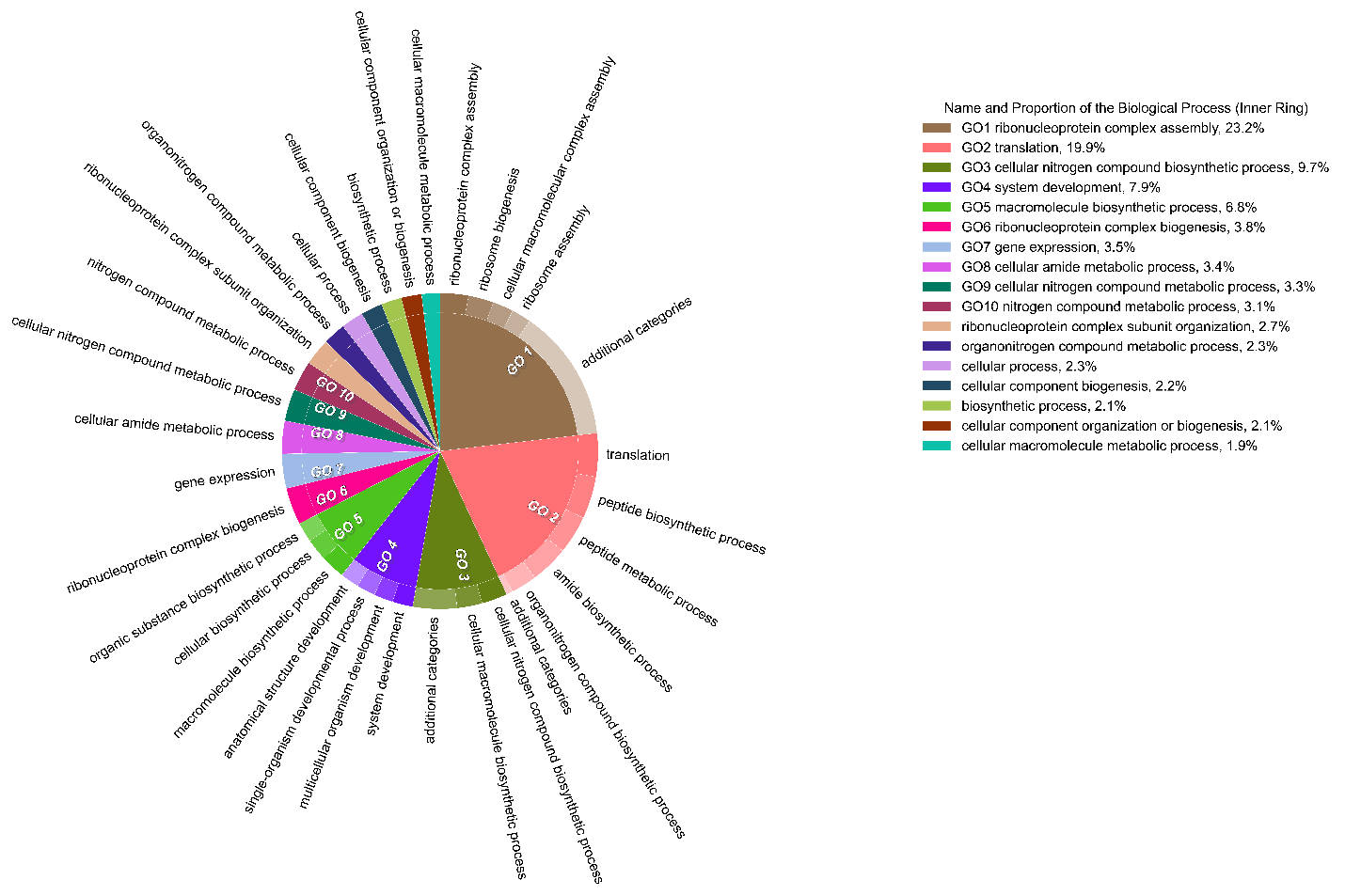
**

**Supplemental Figure 4. Gene Ontology analysis of mid-regeneration timepoints.** Gene Ontology (GO) was performed on the time-course analyses performed on the mid-regeneration (72hpl, 5, and 10dpl) subgroup of the eight original time points. All 350 GO terms with p<0.05 were run through the “Reduce and Visualize Gene Ontology” (REVIGO) software to remove redundant GO terms based on similarity. Circular Visualization plots were generated using the Circular Gene Ontology terms Visualisatio­n (CirGO) algorithm displaying up to 20 of the most represented categories. Inner rings represent the hierarchical summary categories identified by the REVIGO software that contain the subcategories labelled in the outer rings. Keys below each graph represent labels for the inner ring categories. Subcategories within each inner-ring hierarchy are displayed as text in the outer ring. The mid-regeneration plot contains many of the same transcription and translation processes as the early time points with an emphasis on RNA processing and the emergence of developmental processes.

**
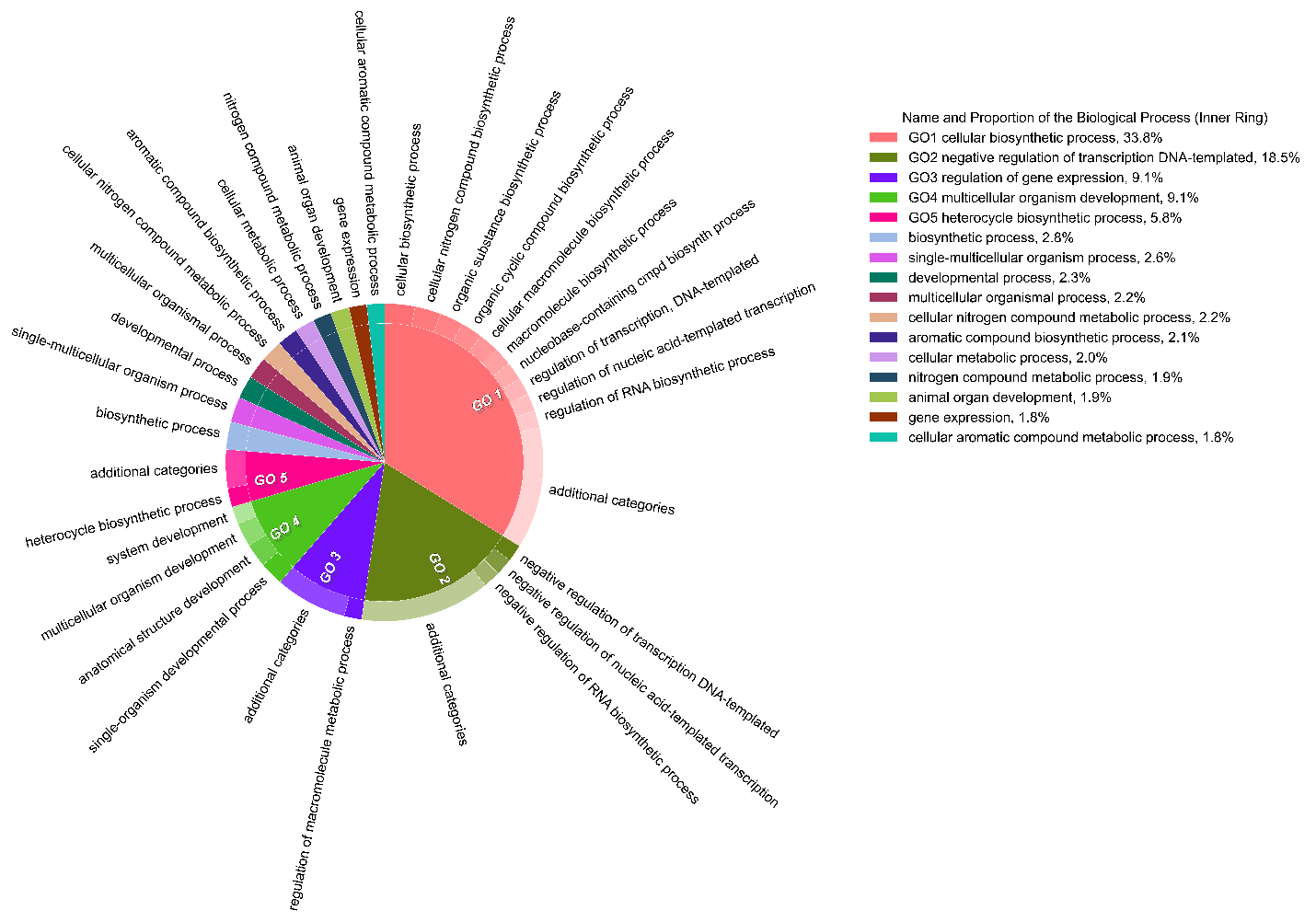
**

**Supplemental Figure 5. Gene Ontology analysis of late regeneration timepoints.** Gene Ontology (GO) was performed on the time-course analyses performed on the late regeneration (10, 14, and 28dpl) subgroup of the eight original time points. All 96 GO terms with p<0.05 were run through the “Reduce and Visualize Gene Ontology” (REVIGO) software to remove redundant GO terms based on similarity. Circular Visualization plots were generated using the Circular Gene Ontology terms Visualisation (CirGO) algorithm displaying up to 20 of the most represented categories. Inner rings represent the hierarchical summary categories identified by the REVIGO software that contain the subcategories labelled in the outer rings. Keys below each graph represent labels for the inner ring categories. Subcategories within each inner-ring hierarchy are displayed as text in the outer ring. Late time points represent a shift from producing gene expression machinery towards biosynthesis, regulation, further developmental processes, and importantly, negative regulation.

**Supplemental Table 1.** [File name > Table-1_Kramer_2021]

This table contains mapped read pseudocounts for all samples collected in this manuscript as well as pairwise comparison statistics for the dark-adapted 0hr dataset to each of the 7 tissue collection time-points throughout the time-course.

**REFERENCES**

1. Thomas, J.L., et al., *Reactive gliosis in the adult zebrafish retina.* Exp Eye Res, 2016. **143**: p. 98-109.
